# Protein–Ligand Interactions in Scarcity: The Stringent Response from Bacteria to Metazoa, and the Unanswered Questions

**DOI:** 10.3390/ijms24043999

**Published:** 2023-02-16

**Authors:** Sailen Barik

**Affiliations:** EonBio, 3780 Pelham Drive, Mobile, AL 36619, USA; eonbiohelp@gmail.com

**Keywords:** protein–ligand, stringent response, magic spot, ppGpp, Mesh1, RelA, starvation, interactome, DksA

## Abstract

The stringent response, originally identified in *Escherichia coli* as a signal that leads to reprogramming of gene expression under starvation or nutrient deprivation, is now recognized as ubiquitous in all bacteria, and also as part of a broader survival strategy in diverse, other stress conditions. Much of our insight into this phenomenon derives from the role of hyperphosphorylated guanosine derivatives (pppGpp, ppGpp, pGpp; guanosine penta-, tetra- and tri-phosphate, respectively) that are synthesized on starvation cues and act as messengers or alarmones. These molecules, collectively referred to here as (p)ppGpp, orchestrate a complex network of biochemical steps that eventually lead to the repression of stable RNA synthesis, growth, and cell division, while promoting amino acid biosynthesis, survival, persistence, and virulence. In this analytical review, we summarize the mechanism of the major signaling pathways in the stringent response, consisting of the synthesis of the (p)ppGpp, their interaction with RNA polymerase, and diverse factors of macromolecular biosynthesis, leading to differential inhibition and activation of specific promoters. We also briefly touch upon the recently reported stringent-like response in a few eukaryotes, which is a very disparate mechanism involving MESH1 (Metazoan SpoT Homolog 1), a cytosolic NADPH phosphatase. Lastly, using ppGpp as an example, we speculate on possible pathways of simultaneous evolution of alarmones and their multiple targets.

## 1. Introduction

In order to survive in stressful conditions, living cells have evolved mechanisms to sense the stress and synthesize specific small molecules that are generally termed as ‘second messengers’. In the bacteria, common such messengers are specialized purine nucleotides, such as cyclic AMP and GMP (cAMP, cGMP, respectively), cyclic di-GMP and di-AMP (c-di-GMP, c-di-AMP, respectively), and the guanosine tetra- and penta-phosphates, collectively denoted as (p)ppGpp [1]. Each of them acts as a ligand for a specific family of cellular proteins, activating physiologically important signaling networks. The eukaryotes synthesize cyclic nucleotides such as 2′,3′-cGAMP from ATP and GTP, but it is primarily dedicated to innate immunity against pathogenic molecules [2,3,4]. The (p)ppGpp pair was discovered nearly half a century ago in nutrient-starved *E. coli* cell extracts, as novel spots on a thin-layer chromatogram, hence named ‘magic spots’ [5,6,7,8]. An explosion of subsequent research revealed the mechanisms of (p)ppGpp synthesis and degradation, and the multiplicity of cellular genes regulated by (p)ppGpp, which eventually leads to survival during stress. Accumulated evidence suggests that the stress response entails a large web of regulatory circuits, perhaps underscoring its importance in cellular and organismic persistence. As indicated earlier, our knowledge on (p)ppGpp derives overwhelmingly from bacteria (mainly *E. coli* and *B. subtilis*), although limited evidence of these nucleotides in selected metazoa (such as *Drosophila*, lower plants, and human) has emerged recently [9,10,11,12,13,14,15]. The Archaea is generally considered devoid of a canonical stringent response, as the search for (p)ppGpp in this kingdom has produced conflicting results [16,17,18].

## 2. Plurality of ppGpp Synthetic and Homeostatic Pathways

**Figure 1:**
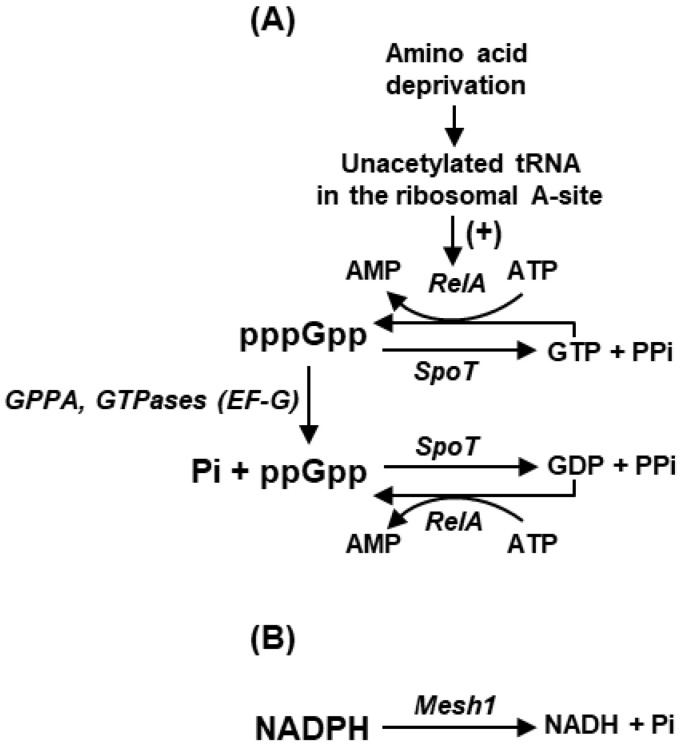
Major pathways of metabolism of nucleoside phosphates relevant in stringent response. (**A**) The synthesis of the two guanosyl phosphates, ppGpp and pppGpp, in bacteria is catalyzed in steps of phosphorylation and dephosphorylation, as shown, using GDP and GTP as substrates. The phosphorylation reactions are reversed by the hydrolase activity of SpoT. Conversion of pppGpp to ppGpp is also catalyzed by various hydrolytic enzymes, such as guanosine pentaphosphate phosphohydrolase (GppA) and Elongation Factor G. (**B**) In the ‘stringent-like’ response in the metazoa, NADPH is dephosphorylated by MESH1, a mammalian SpoT homolog, and can be resynthesized by phosphorylation with NAD kinase (not shown here) (details of MESH1 in Section 4).

Nutrient deprivation, commonly referred to as the ‘stringent response’, is the originally discovered signal of (p)ppGpp synthesis [5,19]. In the early experiments, the pathway was routinely induced by shifting *E. coli* cells from rich media such as the customary Luria Broth (with glucose) to synthetic minimal media such as M9, devoid of amino acids. The (p)ppGpp synthesis is catalyzed by bifunctional proteins, RelA and SpoT, which use ATP and GTP/GDP as substrates; together, these two enzymes comprise the founding members of the eponymous RelA-SpoT homolog (RSH) family [17]. The members of this family share domain structures, notably a (p)ppGpp synthesis (SYNTH) and a (p)ppGpp hydrolysis (HD) domain; however, RelA is responsible for the bulk of synthesis, whereas SpoT is more involved in (p)ppGpp hydrolytic degradation (Figure 1). This is because the SYNTH domain of SpoT has very weak activity, and reciprocally, the HD domain of RelA has no degradative activity.

In the predominant stringent response mechanism, when amino acids are in short supply, RelA detects the 3′-CCA terminus of non-acylated tRNA; the nucleoprotein complex then loads onto the ribosomal A-site of the translating ribosome, and the resultant structural changes preclude the binding of aminoacylated tRNA [20,21,22]. Simultaneously, the interaction changes the RelA structure, activating its (p)ppGpp synthesis activity [23,24,25,26,27].

In general, the RelA, SpoT, and several other players regulate the steady-state level of (p)ppGpp in the bacterial cell, maintaining strict homeostasis in response to changing nutritional conditions, i.e., not only raising (p)ppGpp levels in nutrient deficiency but also bringing it down to low basal levels when nutrients become available [28,29,30]. Precise tuning of the opposing synthetic and hydrolytic activities also allow the cell to achieve a continuum of (p)ppGpp concentrations ranging from micromolar to millimolar, which suits the exact degree of nutritional deficiency and the diverse affinity of the (p)ppGpp-binding proteins [31,32]. Besides SpoT, two other notable degradative enzymes include guanosine pentaphosphate phosphohydrolase (GppA) and several GTPase enzymes, such as the GTPase activity of the translation Elongation Factor EF-G (Figure 1) [33].

Intriguingly, a plethora of other deficiencies and stress conditions also activate (p)ppGpp synthesis; they include, but are not limited to, fatty acid limitation [34], temperature shift [35,36], carbon [37,38] and phosphate [39] starvation, and iron limitation [40]. While RelA is the canonical enzyme for (p)ppGpp synthesis in amino acid starvation, SpoT is capable of mediating (p)ppGpp synthesis in response to these other signals, namely carbon, iron, phosphate, and fatty acid deprivation [34,40,41,42]. The mechanisms of SpoT activation are relatively unclear, but direct interactions with cytosolic accessory proteins appear to modulate the ratio of its synthetase (also termed ‘synthase’) versus hydrolase activities. Fatty acid deficiency, for example, is sensed by the Acyl Carrier Protein (ACP), a central cofactor in fatty acid biosynthesis, which also physically binds to SpoT but not RelA. The binding has an allosteric effect on the SpoT structure such that its (p)ppGpp synthesis domain is activated and the degradative domain is suppressed [34,40]. Lastly, the quantitative aspects of the stringent response, often overlooked, add additional layers of complexity to the mechanism; for instance, early studies in *Bacillus stearothermophilus*, a thermophilic bacteria, revealed that the amount of (p)ppGpp synthesized depends on the nature of the trigger [43]. Specifically, when the deficiency of tRNA acylation is the trigger, (p)ppGpp reaches a much higher level than in carbon starvation or temperature downshift. Clearly, the signaling pathways that regulate (p)ppGpp homeostasis differ among the triggers, perhaps due to the involvement of different (p)ppGpp synthetase paralogs [43]. Further studies may include a combination of two triggers to see if they produce an additive effect on (p)ppGpp synthesis or whether one trigger dominates the other.

Due to the diverse nature of these sensing mechanisms, we only mention them briefly under ‘Unanswered questions’ (Section 5) while the bulk of the review remains focused on the classical stringent response, triggered by amino acid starvation. Various other molecular mechanisms of RSH regulation, such as the complex inter- and intra-molecular interactions between the domains of Rel and SpoT, are also outside our scope but can be found in several research papers and are also cited in recent reviews, such as Sinha and Winther, 2021 [44].

## 3. The Complex Network of (p)ppGpp Signaling

Like all second messengers, the stress-generated (p)ppGpp regulates a bewildering number of signaling steps and pathways, affecting multiple aspects of cellular metabolism and behavior, reported and reviewed by many leading investigators over the years [1,31,45,46,47,48,49,50,51,52,53]. The majority of the effects can be explained by the need for activation of emergency functions required for survival during stress, while conserving the cellular resources by shutting down housekeeping genes that are dispensable for the short term. The latter class includes relatively stable macromolecules, such as transfer RNA, ribosomal RNA, and a large number of proteins. Often, inhibition by (p)ppGpp involves direct binding to its target protein in a domain that normally binds GTP in the unstressed cell. This section provides a brief but mechanistic summary of the different modes and steps of the regulations.

### 3.1. Transcriptional Regulation

One of the earliest discovered effects in the stringent response was selective induction and suppression of bacterial transcriptional promoters [19,45,54,55,56,57], which was further confirmed and extended by subsequent transcriptome analysis [58,59,60,61,62]. Multiple mechanisms have been detected for (p)ppGpp-mediated inhibition of transcription, with direct binding to RNA polymerase (RNAP) being the most straightforward among them, as shown for *E. coli* [63,64]. Here, instead of binding at the catalytic center or the nucleotide-binding site, (p)ppGpp binds at two regions, one at the interface between the RNA polymerase (RNAP) β’ and ω subunit, and the other between RNAP and the transcription initiation factor, DnaK suppressor A (DksA) [63,64,65,66,67,68]. Naturally, the relative binding of (p)ppGpp to the two sites depends on DksA concentration in the cell, such that they function coordinately in different nutritional or environmental conditions [68,69,70]. Based on several lines of in vivo and in vitro results, these authors concluded that Site 1 would be preferred when ppGpp concentrations are low, i.e., in nutrient-rich medium, and Site 2 would be preferred when ppGpp concentrations are high enough to fill both sites. This is a noteworthy example of a functional allosteric switch operated by the same ligand binding at two different locations of the same enzyme [71,72]. In the other mechanism, (p)ppGpp inhibits the binding of the housekeeping sigma factor (σ70) to the RNAP [73], which allows the RNAP to use σ32 and σ38, two alternative sigma factors that are specific for the promoters of genes induced during stress and the stringent response [73].

In Gram-positive bacteria, exemplified by *B. subtilis*, the mechanism is entirely different and more indirect; here, instead of binding to RNAP, (p)ppGpp directly binds to the active site of GMK (guanylate kinase), and the resultant inhibition of GMK reduces the GTP pool [46,47,74]. This lowers the transcription from ribosomal RNA promoters, as they initiate with GTP; however, transcription from amino acid biosynthesis operons is induced, mainly because CodY, the repressor of these operons, requires GTP to repress their promoters. Besides *B. subtilis*, this mechanism was evidenced in several other bacteria, including *Thermus thermophilus*, a Gram-negative, thermophilic bacterium [75]. A survey of GMKs from phylogenetically diverse bacteria indeed revealed that the mechanism is conserved in the Actinobacteria, Firmicutes (e.g., *B. subtilis*), and Deinococcus-Thermus, whereas, in the Proteobacteria (e.g., *E. coli*), (p)ppGpp directly regulates RNA polymerase (RNAP) [47]. The authors proposed that GMK is an ancestral (p)ppGpp target, whereas RNAP evolved as a direct target in Proteobacteria only recently [47].

As the direct interaction of (p)ppGpp with RNAP occurs via binding to the interface of β’ and ω, it is in effect a tripartite interaction involving β’, ω, and ppGpp, which complicates the structure–function correlation studies. In an example, a high-resolution co-crystal structure identified a ppGpp-binding pocket on Thermus thermophilus RNAP [76]. In a complementary study [77], genome-wide transcription initiation sites in *E. coli* cells were mapped by RNA-seq analysis under two conditions: low basal levels of intracellular ppGpp and at higher levels, produced by expression of recombinant RelA, which allowed studies of the role of ppGpp in the absence any indirect effect of stress. The results suggested that ppGpp indeed directly interacts with RNAP to transcribe different populations of genes in low and high ppGpp environments, commensurate with the use of two different ppGpp binding sites. Surprisingly, mutations of the ppGpp contact resides on the RNAP failed to abrogate the ppGpp effect, as measured in well-defined RNAP-promoter interactions [75]. Evidently, multiple lines of structural and biochemical evidence need to be pursued to determine whether the ppGpp-regulation of transcription occurs through direct interaction, or indirectly, via other proteins (such as DksA).

#### DksA and GreA/B: An Unexpected Similarity

**Figure 2:**
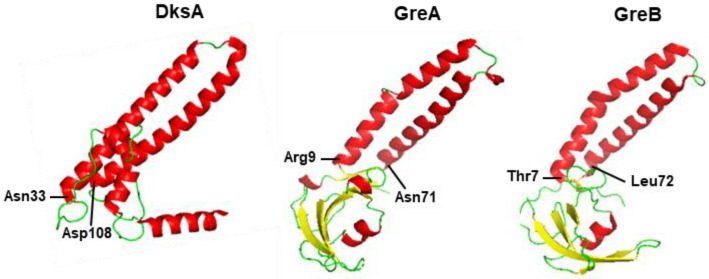
Structure similarity between DksA and GreA/B proteins. The coil-loop-coil domain of the proteins are shown, using the PyMol software [88]. The structures, fetched from the open-access RCSB PDB (Research Collaboratory for Structural Bioinformatics Protein Data Bank) at www.rcsb.org, are: 1TJL (DksA), 1GRJ (GreA), and 2P4V (GreB). The amino acid residues at the beginning and end of the domains are labeled; the helices are colored Red, whereas the loop regions and beta-strands are Green and Yellow, respectively.

The transcriptional elongation complex is regulated by a large number of protein factors that control pause and termination, which have been studied in great detail in *E. coli.* As implicated earlier, the *E. coli* RNAP encompasses two spaces, discernible in its 3D structure, referred to as primary and secondary channels, as recently summarized [78]. While the primary channel binds the template DNA and is the site for nascent RNA synthesis, the secondary channel serves as the entrance for ribonucleotides and for the elongation regulatory factors, such as the DksA and ppGpp. Biochemical and genetic analyses, independent of the stringent response, revealed the existence of protein factor(s) that suppress transcriptional pause. Initially, the use of transcription reactions in vitro showed that cell-free *E. coli* lysates kinetically suppress the +15/+16 pause at the late promoter (pR’) of coliphage lambda without affecting the termination property of the polymerase [79]. Subsequently, a flurry of elegant studies characterized two highly homologous proteins, named GreA and GreB, as pause suppressors [80,81,82,83]. In parallel, structural studies showed that all three proteins (GreA, GreB, DksA) contain large coil-loop-coil (also known as ‘coiled-coil’) domains (Figure 2) that interact with the secondary channel of the *E. coli* RNAP and make specific contacts, which may underlie the observed competition among them [84,85,86,87]. The commonality of structure as well as binding of DksA and GreA/B to the same RNAP channel is a remarkable discovery that may have important ramifications on the possible competition between them, which remains to be explored [78].

### 3.2. Regulation of Translation by (p)ppGpp

In the stringent response, it is logical to assume that the shortage of amino acids would lead to the immediate and drastic reduction in protein synthesis. To this end, (p)ppGpp shuts off translation by directly binding to and inhibiting multiple components of the translation machinery [45,53]. The bacterial translation initiation factor IF2, for instance, uses GTP in the formation of the 30S initiation complex; this is inhibited by high concentrations of ppGpp as the latter competes out GTP [89,90,91]. However, translation of selective mRNAs with distinctive hairpin structures can still occur, as, in these cases, IF2 is able to use ppGpp to initiate protein synthesis [91]. The mechanism of how the hairpins allow 30S assembly by IF2-ppGpp remains to be determined. (p)ppGpp also binds the elongation factors, EF-Tu and EF-G, and affects the translation rate in a complex manner that depends on its relative concentration and other translational factors [92]. Finally, (p)ppGpp inhibits a large number of known and putative GTPases, required for the biosynthesis and maturation of bacterial ribosomes, such as RsgA, RbgA, Era, HflX, and Obg [93,94].

**Figure 3:**
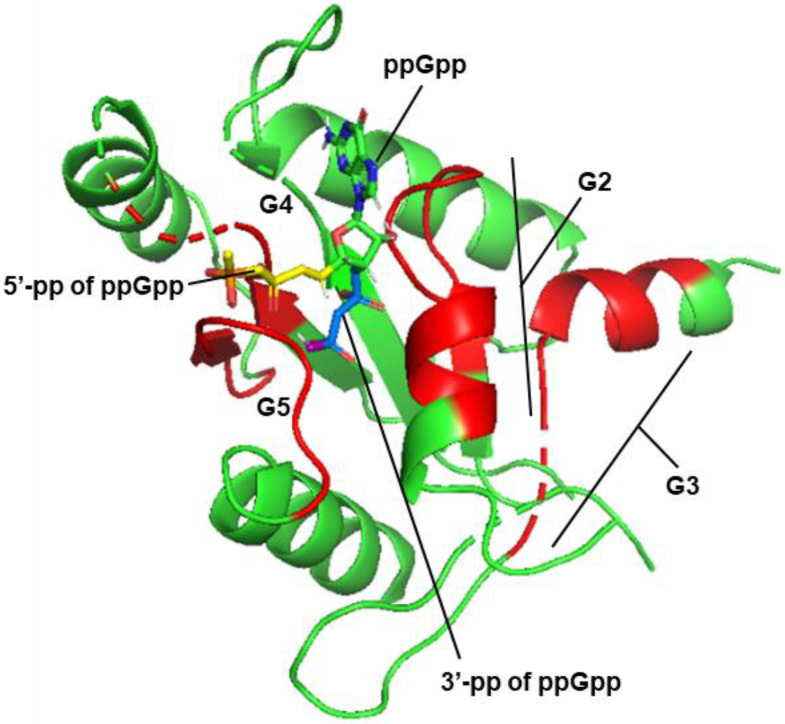
In silico docking of ppGpp onto *B. subtilis* Obg (PDB 1LNZ). This presentation was generated by PyMol software [95] of the docked complex created by the CB-Dock2 software [96], based on a cavity search followed by optimal ligand-fit, the details of which have been described (Supplementary material). Much of the protein structure has been removed in the display for better visualization of ppGpp-binding, as labeled. The four areas colored red, and designated as G2, G3, G4, G5, are conserved in guanine nucleotide-binding proteins [31,94] and are also close to the docked ppGpp molecule. The 5′ and 3′ diphosphate chains of ppGpp are colored orange and blue, respectively. The rest of the polypeptide, regardless of secondary structure, is green.

Experimentally determined crystal structures and docking by Molecular Dynamics simulation previously showed that (p)ppGpp indeed occupied the GTP-binding domain of these proteins [31,95]. Of these short-listed proteins, the Obg protein of *E. coli* (ObgE) has several important roles, including interactions with ppGpp and with the peptidyl-transferase center, and maturation of the 50S ribosomal subunit [95]. As one of our goals was to study protein–ligand interactions in the stringent response by in silico analysis, as presented in the later sections, here, we tested selected docking programs for their conformity with the published 3D structure of the complexes, obtained from the structure databank (RCSB PDB). We used the CB-Dock2 program for this purpose [96], as described in the Supplementary material. Our independently predicted structure (Figure 3) obtained by the docking of ppGpp into the 3D structure of B. subtilis Obg protein, using this program, was also in close agreement with the previous ones [31], confirming that ppGpp docked very close to the active site.

### 3.3. Regulation of Replication by (p)ppGpp

The ability of (p)ppGpp to suppress bacterial cell division was known since the discovery of the stringent response [19]; however, studies of its molecular mechanism have remained scanty. Bacterial DNA replication, essential for cell division, is inhibited by (p)ppGpp both directly and indirectly, depending on bacterial species [97,98]. In *E. coli*, (p)ppGpp inhibits replication initiation through mechanisms that are dependent on hemimethylation of DNA at the origin of replication (OriC) and binding of the SeqA protein, a negative modulator of replication initiation [98,99,100,101]. Recent studies indicated an underlying mechanism based on the coupling of transcription and DNA replication, modulated by DNA supercoiling [102]. In it, a transcription fork in the vicinity of oriC induces negative supercoiling and DNA strand separation at oriC, which, in turn, facilitates initiation of replication. As rRNA constitutes over 80% of all transcription in a bacterial chromosome and the rRNA loci are clustered near the oriC [103], a global inhibition of transcription by ppGpp during the stringent response could, in principle, diminish the introduction of negative supercoils near oriC, also making the oriC more difficult to melt [104,105], leading to the observed inhibition of replication initiation. However, Fernandez-Coll et al. [106] provided a strong argument that regulation by ppGpp does not require the ribosomal operons to be present in cis with oriC; instead, ppGpp alters the expression of gyrase and topoisomerase IV, which is responsible for modifying the supercoiling around the origin.

The stringent response also inhibits DNA elongation [107,108]. While the effect on gyrase and topoisomerase may also underlie the inhibition, a large body of evidence has established a direct inhibition of DNA primase (DnaG) by (p)ppGpp [107,108,109,110,111]. DNA primase is a specialized RNA polymerase that catalyzes the synthesis of short RNA molecules, ~11 nt long, used as primers by DNA polymerase at the replication forks in all stages of replication [112,113,114]. The (p)ppGpp binding site of primase overlaps with the NTP binding site, and therefore, (p)ppGpp competes with NTP for binding. However, the primase is inhibited by low concentrations of ppGpp (<1 mM) [31,68] that may be attained early in the stringent response, paralleling the rapid cessation of DNA replication and cell division, further implicating the primase as a key player in the ppGpp effect. Lastly, (p)ppGpp appears to destabilize the association between primase and the RNA-DNA heteroduplex [108].

### 3.4. The (p)ppGpp Interactome

Since the recognition of the stringent response, nearly two dozen ppGpp-interacting proteins have been reported in focused studies of specific effects [53], a few examples of which such as RNAP (Section 3.1), several translational factors (Section 3.2), and DNA primase (Dna G) (Section 3.3) were presented in previous sections, but broader studies revealing many other interactors are discussed here. In an unbiased effort to identify ppGpp-binding proteins, two groups used differential-radial-capillary-action-of-ligand-assays (DRaCALAs) that essentially measure the mobility of radiolabeled ppGpp on a nitrocellulose filter treated with lysates of cells overproducing a single candidate protein [115]. DRaCALA led to the identification of a small number of targets, a total of ~30 in Staphylococcus and *E. coli* added together [115]. This was an encouraging proof-of-concept as the identified candidates included several bona fide proteins, such as five GTPases involved in ribosomal biogenesis and nine known ppGpp-binding proteins [115]. However, if one appreciates the vast functional network of the ppGpp effect, one would perhaps expect a larger number of ppGpp-binding proteins. In fact, as pointed out [116], DRaCALA had missed several proteins that were known to bind ppGpp in vitro, which was conjectured to result from the fact that being a non-equilibrium assay, DRaCALA missed targets with fast off-rates, i.e., some filter-immobilized proteins may have lost the radioactive ppGpp faster than they could bind. Moreover, the need for purified recombinant proteins, which is labor-intensive, limited the number of proteins screened. Recently, multiple additional ppGpp-binders were identified in *E. coli* by photoaffinity capture [117]. In this method, trifunctional photoaffinity ppGpp compounds are first synthesized, in which ppGpp is coupled with biotin and phenyl azide. The compounds are incubated with extracts of cells that underwent the stringent response, and cross-linking by UV light leads to formation of the covalent bond that prevents dissociation of the proteins. The captured proteins are separated from the lysate by the use of streptavidin-coated magnetic beads. In the last step, the proteins are digested with trypsin and peptides are characterized by standard LC–MS/MS (Liquid Chromatography-Tandem Mass Spectroscopy) to identify the proteins. As a routine practice in such studies, the proteins are finally identified by using the peptide sequences as a query against the genome-wide proteome.

In a fundamentally similar approach but a more comprehensive expedition, Wang et al. [116] used a similar photoaffinity capture procedure and cross-linkers of different lengths to identify 56 *E. coli* proteins that directly bind (p)ppGpp, including the previously known ones and several new ones. Interestingly, it also appeared to have missed a few known ppGpp-interactors, such as DNA primase (Section 3.3), the reasons of which are unclear. Nonetheless, several of the newly identified proteins were independently confirmed previously by computer-generated docking [31]. Here, we have paid attention to a few of the remaining hits for which, to the best of our knowledge, in silico protein-ppGpp docking studies have not been reported. The goal here was to obtain structural confirmation of some of the biochemical properties of these proteins, where known, and therefore, we used the same CB-Dock2 program (Supplementary material) that we had used earlier. A cautionary note here is that conclusions derived from in silico structures alone may not be fully reliable; however, they can serve as guides for future research.

#### 3.4.1. SpeC, a Constitutive Ornithine Decarboxylase

**Figure 4:**
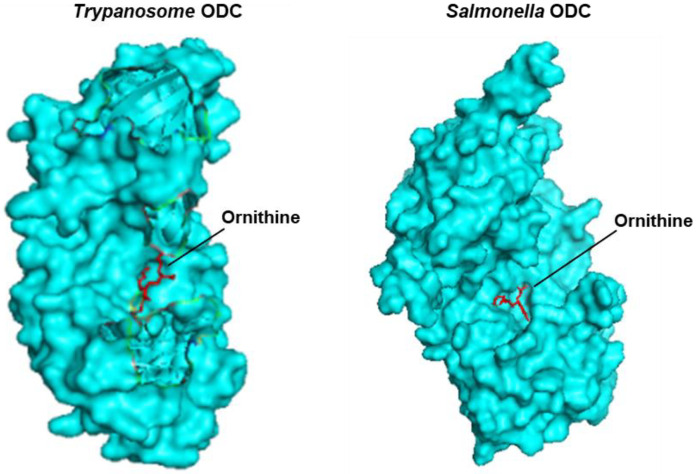
Structural comparison between ornithine docked into *S. typhimurium* ornithine decarboxylase (ODC) and the crystal structure of ornithine-*T. brucei* ODC complex, using CB-Dock2 (Supplementary material). The surface topology view of each structure, presented in PyMol (cyan color), shows the ornithine molecule (in red) in a crevice at approximately similar locations in the overall structure.

One of the hits in the ppGpp-affinity Mass-Spec described above is SpeC, a ‘constitutive’ ornithine decarboxylase (ODC) in *E. coli* (UniProtKB P21169) [118]. However, its three-dimensional structure is not available. The structure of the D-ornithine decarboxylase of Salmonella [119] (SpeF; UniProtKB Q8ZNC4), also a Gram-negative bacterium and phylogenetically very close to *E. coli* [118,119], is known (PDB 6N2H), but it also decarboxylates D-lysine, and uses both amino acids in D-configuration rather than the usual L form [120]. At a length of 465 amino acids, it is also much shorter than E. coli SpeC (711 amino acids). The structure of the other major ODC (SpeF) of *E. coli*, which belongs to the ‘inducible’ class of ODC, has recently been solved, but in complex with stalled ribosomes [121]. On the other hand, the apo structure of Salmonella SpeF is available for the single polypeptide (PDB 6N2H). Lastly, the structure of a 445 aa long ODC form Trypanosoma brucei (XP_011780677.1, PDB 1NJJ) in complex with D-ornithine is available, which is a dimer, but each monomer has a molecule of D-ornithine embedded in it [122]. Its primary structure is highly similar to that of the Salmonella enzyme (Supplementary material), and, thus, we reasoned that the two enzymes may share similar catalytic domains and structures. For the best approximation in the docking analysis, we first docked the structure of ornithine, the substrate, into the available Salmonella SpeF structure (PDB 6N2H), and we found that the resultant structure was comparable to those of the known co-crystal structure of ornithine and the T. brucei enzyme monomer (PDB 1NJJ) (Figure 4). In both cases, ornithine fitted into a bowl-like cavity, similarly located in the 3D structure of the two proteins.

**Figure 5:**
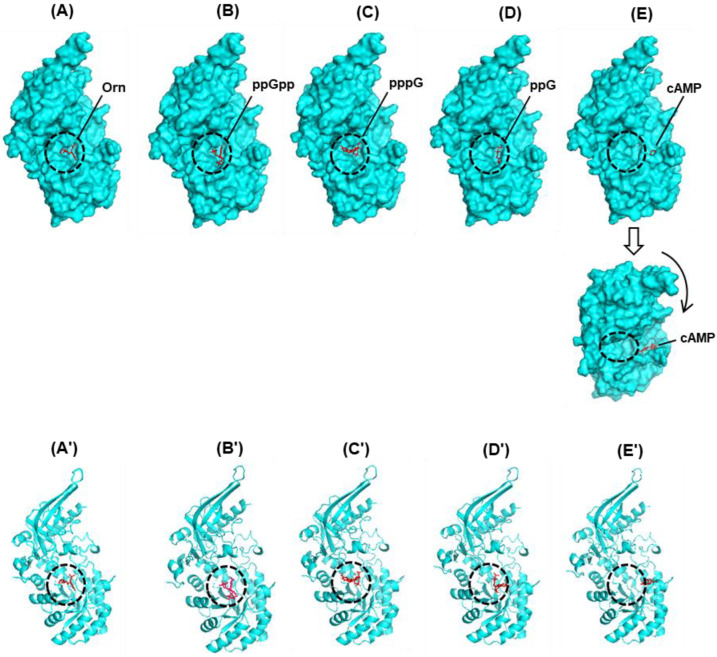
In silico docking of ornithine (**A/A’**), GTP (**B/B’**), GDP (**C/C’**), and cAMP onto the ODC crystal structure (PDB 6N2H) using CB-Dock2 (Supplementary material), as described for Figure 3. All these ligands, colored red, sit in the same cavity that is circled for easy viewing. The docked structures are depicted by PyMol, in surface (**A**–**E**) as well as in ribbon (**A’**–**E’**) presentation. As the cAMP is almost fully hidden when the surface presentation is shown from the same angle as the others, this particular structure is also shown from a different angle after rotation (curved arrow), which reveals how far the cAMP docking site is from that of the other ligands, as it is practically outside the circled cavity.

Some of the earliest biochemical studies of ODC showed that both GTP and ppGpp regulate ODC enzymatic activity [123,124]. Subsequently, a more detailed analysis of partially purified *E. coli* ODC confirmed these results [125], and also showed that both ppGpp and GTP are competitive with respect to ornithine, but GDP or cyclic 3′,5′-AMP (cAMP) had no detectable effect. However, it should be noted that the authors did not know whether the measured activity was due to the constitutive enzyme (SpeC) or the inducible one (SpeF) [125]. Starting with the same ODC structure (PDB 6N2H), we conducted in silico docking of four different ligands to it: ornithine, ppGpp, guanosine 5′-triphosphate (GTP or pppG), guanosine 5′-diphosphate (GDP or ppG), and cAMP. As shown by the same scale of presentation (Figure 5), all ligands bound inside or near the same cavity; interestingly, however, ppGpp and GTP were positioned very close to the ornithine-docking site, whereas GDP and cAMP were considerably farther, with cAMP being the farthest.

In fact, the cAMP molecule is so far away that it was deep inside a small tunnel to the far right corner of the cavity (Figure 5E,E’) and was barely visible in this angle of presentation; to make it easily visible, we made a second presentation, in which the complex is rotated downward. For better viewing of the interior of the complexes and the structural segments, the same molecules are also presented in a ribbon diagram, where the comparative distances are clearly discernible (Figure 5A–E). These results may explain why ppGpp and GTP in the previous study competed with ornithine for interaction with ODC, while GDP and cAMP showed no detectable effect [125]. However, it should be noted that the actual mechanism may be more complex, as ODC regulation is affected by multiple ligands binding at various allosteric sites with different affinities [123,124,125,126,127].

#### 3.4.2. Uracil Phosphoribosyltransferase

As an important enzyme for transcription, uracil phosphoribosyltransferase (UPRT) catalyzes the reaction: uracil (U) + phosphoribosyl pyrophosphate (PRPP) → uridine monophosphate (UMP) + pyrophosphate (PPi) [128]. The hydrolysis of the high-energy phosphodiester bond of the inorganic pyrophosphate (PPi) drives the otherwise reversible reaction to the right, resulting in the synthesis of UMP, a ubiquitous starting material for the cellular pyrimidine biosynthetic pathway. It was one of the major enzymes detected in the ppGpp affinity screening [116], suggesting that it may be inhibited in the stringent response, when replication and most of the transcription are halted as mentioned before, and therefore, nucleotide use is greatly reduced (Section 3.1 and Section 3.3).

**Figure 6:**
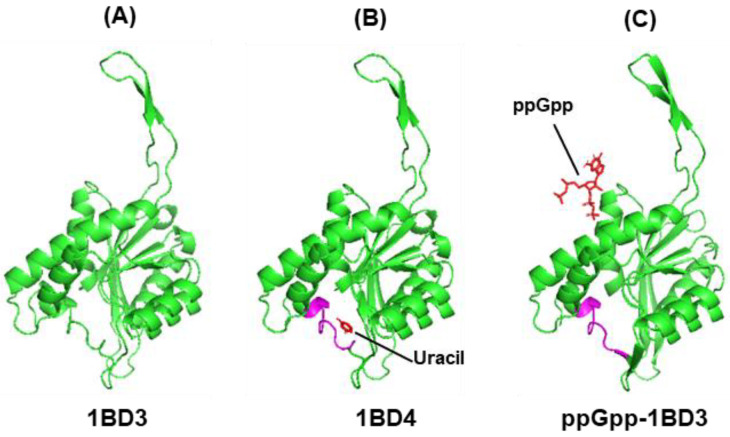
In silico docking of ppGpp to UPRT. The crystal structures of the apo enzyme (**A**) and its complex with uracil, the substrate (**B**), are, respectively, PDB 1BD3 and PDB 1BD4, as shown. The in silico docked co-complex of 1BD3 and ppGpp (**C**) show the significant difference in binding sites of the two ligands (both in red color). The pink segment, spanning a loop and a short helix, represent a region that was considered important for interaction with uracil [128,129,130]. Structure-blind docking was performed by CB-Dock2 (Supplementary material) as described earlier.

To our knowledge, the 3D structure of *E. coli* UPRT is currently not available; however, those of Toxoplasma gondii (PDB 1BD3) and Mycobacterium tuberculosis (5E38) are known and are very similar [128,129]. The amino acid sequences of all three are also highly similar, particularly in the C-terminal half. Of these, we chose the T. gondii enzyme because its structure has been solved not only in the apo form (PDB 1BD3), but also in complex with uracil (PDB 1BD4) (Figure 6A,B).

We first ran CB-Dock2 on ppGpp-1BD3 and ensured that the in silico structure is comparable to that of the actual crystal structure, 1BD4 (Figure 6A,B). We note that UPRT is a complex enzyme that can exist in multiple conformations, and it belongs to the ‘morpheein’ class of proteins that can form multiple different oligomers by changing the shape of the monomer [131]. In both T. gondii and M. tuberculosis, the UPRT structure is a symmetrical tetramer [128,129]. In T. gondii UPRT, each monomeric unit binds a uracil in an identical position to produce 1BD4 [128]. In contrast, the *E. coli* enzyme has been reported to be a trimer [130]. In order to avoid the complication of diverse multimers, we remained focused on a single, monomeric polypeptide. Docking ppGpp onto 1BD3 showed that unlike in ornithine decarboxylase (Section 3.4.1), it did not dock in the substrate (uracil)-binding pocket, but docked in a completely different area (Figure 6, compare between Panels B and C). In fact, it is possible that in 1BD3, ppGpp binds to the interface region between two monomers, contacting both. A detailed analysis of the binding mode of ppGpp should await actual crystal structures. Nevertheless, these studies will be relevant for M. tuberculosis as well, as this important human pathogen also has a robust stringent response regulated by ppGpp, reviewed recently [132].

Together, many elegant structural analyses of the past [31] and our in silico docking of two high-scoring ppGpp-binders [116] reveal the abundant variety of ppGpp targets and the binding patterns, explaining the large network of pathways triggered in the stringent response.

## 4. Metazoan ‘Stringent-like Response’

**Figure 7:**
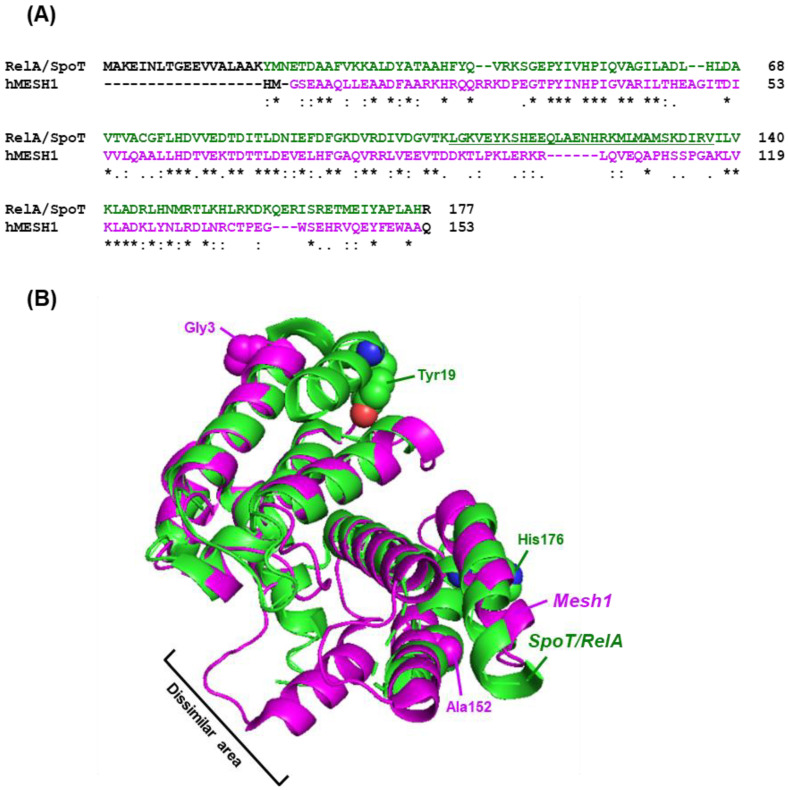
Sequence and structure homology between representative bacterial RelA/SpoT and human MESH1. The homologous regions of the two amino acid sequences, aligned by Clustal Omega (**A**), and the PDB structures of the same regions, aligned and superimposed by PyMol (**B**), are shown for the RelA/SpoT protein of the Gram (+)ve bacterium, *Streptococcus equisimilis* (PDB 1VJ7; 2.1 Å resolution) (Green color) and human MESH1 (PDB 5VXA; 2.1 Å resolution) (Magenta color). By the standard convention of Clustal alignment, the asterisk (*), colon (:), and period (.) indicate identical, strongly similar, and weakly similar residues, respectively. The total length of the SpoT/RelA protein is 387 amino acids, while the MESH1 protein is 180 residues long, of which only the N-terminal homologous regions are shown in both panels to conserve space. As shown, this region is free of beta strands and made exclusively of alpha helices connected by short loops (Panel **B**), and a dissimilar region of ~30 amino acids (underlined in Panel **A**) separate the two homologous clusters of helices (Panel **B**). Also in panel B, the N-terminal and C-terminal residues of these sequences are indicated, using the same color code as the proteins (namely Tyr19, His176 in Green for RelA/SpoT and Gly3, Ala152 in Magenta for human MESH1).

The small alarmone class of synthetases and hydrolases (SAS, SAH), mentioned earlier, is found in prokaryotes and a few lower eukaryotes, but is absent in the metazoa, including all vertebrates such as humans [17]. In fact, as of this writing, there are no reports of a canonical prokaryote-like stringent response. Nonetheless, it is worth drawing the readers’ attention to the intriguing discovery [133] of a SpoT sequence ortholog in Drosophila melanogaster (the ‘fruit fly’) and in cultured human cells, which was named MESH1 and found to play an important role in nutrient deficiency. The response was dubbed a ‘stringent-like response’ by the authors; however, this response turned out to be fundamentally different from the canonical stringent response in prokaryotes. Also noteworthy is that MESH1 appears to be an ortholog of SpoT in sequence and structure (Figure 7), but not in function, and that it has no synthetase activity [134]. In complementary reports, ppGpp was identified as an alarmone in the fruit fly, in human cells [13], and in a few plant chloroplasts [14,15]. Due to its apparently rare occurrence and under-studied mechanism, the ‘stringent-like response’ is summarized here only briefly.

When expressed recombinantly, the MESH1 protein functionally partially complemented SpoT-deficiency in bacteria and suppressed RelA-induced inhibition of cell growth, the mechanism of which has not been resolved [11]. Drosophila with deleted Mesh1 gene exhibited stunted body growth and sensitivity to starvation. As in the prokaryotic stringent control, when starved for amino acids, the Mesh1-null fly underwent down-regulation of genes related to replication and translation and upregulation of stress-inducible genes.

Studies of the Mesh1 mechanism of action revealed its major distinction from the canonical stringent response in prokaryotes. First, Drosophila as well as human MESH1 was found to dephosphorylate NADPH (Nicotinamide Adenine Dinucleotide Phosphate, reduced form) [134,135,136] to NADH. Thus, MESH1, unlike SpoT/RelA, exhibits a broader substrate specificity, being able to dephosphorylate both ppGpp and NADPH [135]. Second, loss of MESH1, and the resultant loss of the NADPH phosphatase activity of the cell, appears to protect cells from untoward stress, specifically from ‘ferroptosis’ [135,136]. Recently recognized as a new form of cell death, triggered by iron-dependent lipid peroxidation, ferroptosis was later found to be induced by a variety of stressors, including endoplasmic reticular stress (ER stress), a complex signaling network activated by misfolded or unfolded proteins [137,138]. The apparent overlap between cellular responses to various forms of stress has led to the concept of the ‘integrated stress response’ [139].

Meanwhile, phylogenetic sequence analysis led to the discovery of Mesh1-like genes in several bacterial genera, including Cellulophaga, Erythrobacter, and Flavobacterium [140], coding for SpoT orthologs that contained hydrolytic domains. Interestingly, several species of extremophiles that thrive in harsh habitats, such as extremely high or low temperatures and in saline marine waters, belong to these genera [141]. These extremophiles, especially many Flavobacteria, also encode a unique family of immunophilins, named ‘dual-family immunophilin’ (DFI) [142,143]. The DFI, sometimes referred as ‘foldase’, promotes or maintains native folding of proteins, particularly needed in extreme environments in which proteins would otherwise misfold and/or denature [142,143]. With this premise, we would like to propose that MESH1 (or a close ortholog) and the ER stress response serve as major stress cues in the extremophiles, which awaits experimental evidence.

Structural and functional analysis showed that like the prokaryotic enzyme, the MESH1 is highly similar to the N-terminal half of the bacterial SpoT/RelA polypeptide, which contains a ppGpp hydrolysis domain, and as mentioned, MESH1 catalyzes the dephosphorylation of ppGpp [133,134,135]. When we compared the X-ray crystal structures of the hydrolase domains of a metazoan MESH1 with a bacterial SpoT, using the human and *E. coli* proteins as respective prototypes, the structures could not be exactly superimposed (Figure 7). However, the secondary structure elements within the two domains, flanking the dissimilar middle section, were highly similar overall (Figure 7). The RMSD value of the 3D structure alignment was 0.93; this is indicative of significant structural similarity in the homologous regions, particularly considering that the central dissimilar sequence (underlined in Panel A) contained unaligned loops that incurred a penalty in RMSD [144,145]. To sum up, in spite of their functional differences, the bacterial RelA/SpoT and the eukaryotic MESH1 proteins comprise domains that are highly similar. Reciprocally, it is quite likely that the dissimilar central portion is a major reason behind the enzymatic differences of the two proteins.

It is apparent that the placement of the helices in the higher-order structure of these proteins has evolved to accommodate their substrate preferences, i.e., NADPH versus ppGpp, and perhaps also to interact with phylogeny-specific accessory factors and signaling partners in the protein–protein contact domains.

## 5. Unanswered Questions and Future Directions

### 5.1. The ‘Stringentome’

Over the years, large-scale binary protein–protein interactomes have been generated for several model eukaryotes, some of which are known to possess various forms of stress/stringent responses, as in D. melanogaster [146,147] and humans [148,149]. In yeast, interactomes of stress-related regulatory processes have been called ‘StressNet’ [150]. Such studies can be focused on the stringent response pathway proteins, such as RelA and SpoT, and extended to the ppGpp-interactome mentioned earlier [116,117], to generate the complete stringent interactome that may be named ‘stringentome’, following the trend of the ‘omic’ nomenclature. A comparison of stringentomes of different species will then shed light on how the stringent response and its network may have evolved.

### 5.2. Evolution of the Stringent Response in Bacteria

We start with the basic premise that the stringent response is triggered by an effort of the cell to conserve resources and energy by shutting down cellular functions except the ones that are required for viability. Close analogies of systemic shut-down include bacterial sporulation and the hibernation of animals in winter. However, the biosynthesis of each molecule of (p)ppGpp by RelA uses the high-energy phosphate of ATP (Figure 1), while GTP is used by selective GTP-requiring proteins. In the stringent response, the energy contained in GTP is not lost but is masked in the energy-rich β-γ phosphodiester bond in pppGpp.

While all this is energetically sensible, the difficult part is to visualize how the (p)ppGpp interactome developed, i.e., how so may proteins evolved to bind (p)ppGpp, a specialized pair of nucleotides. In some of these proteins, (p)ppGpp binds the existing GTP-binding pocket, and in fact, the two nucleotides often compete with each other for binding, as exemplified by SpeC (ODC) (Section 3.4.1). In the others, (p)ppGpp seems to bind elsewhere, which therefore, newly evolved (de novo). We speculate two possibilities: the second site is a low-affinity GTP-binding pocket that was never recognized as such and fits (p)ppGpp more snugly; or, it has no GTP-binding ability at all, but truly evolved de novo to bind (p)ppGpp. The two models can be distinguished by using site-directed mutant proteins in binding studies in vitro at different concentrations of GTP/(p)ppGpp.

### 5.3. Replacement of Stringent Response by Stringent-like Response

**Figure 8:**
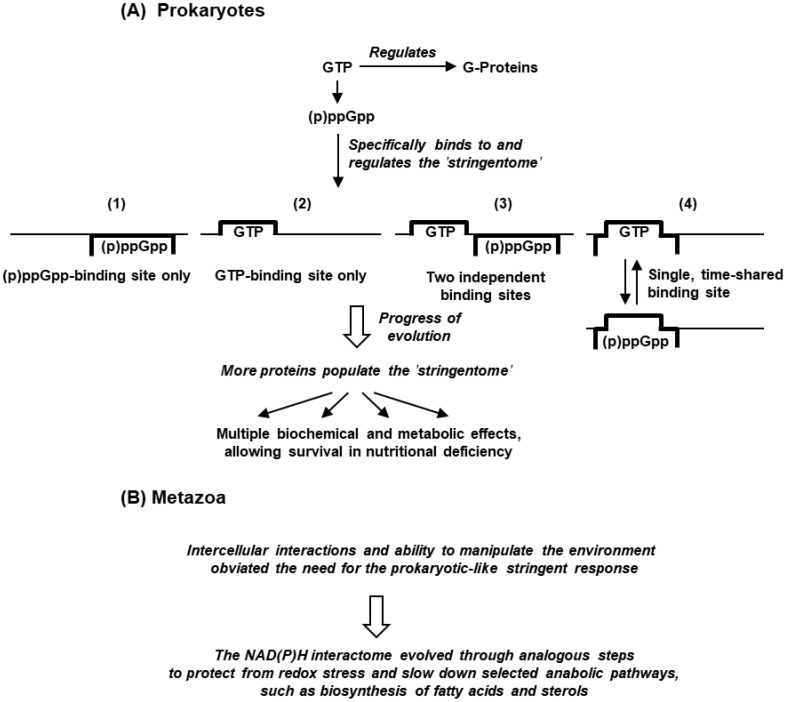
Tentative theory of evolution of the stringent response in prokaryotes (**A**) and the very different response in metazoa, dubbed ‘stringent-like response’ (Section 4) (**B**). This is only a schematic of the biochemical pathways that have been described in this review, integrated with possible steps of evolutionary expansion of the interactome (hereby named ‘stringentome’). In Panel A, we propose four alternative arrangements (1–4) of (p)ppGpp- and GTP-binding pockets in a proximal signaling protein of the interactome, as depicted schematically.

The general disappearance of the classic (p)ppGpp-based stringent response in the evolution of life from unicellular to multicellular organisms is an enigma, but we can try to formulate a scenario based on the genetics and the survival needs of the extant organisms, which is schematically depicted here (Figure 8). It goes without saying that evolutionary models are often fragmented with missing pieces, and the stringent response is no exception. However, we hope that the discussions here will invite refinements, alternative models, and experimental verifications, where possible.

The paucity of (p)ppGpp in the metazoa also coincides with the apparent lack of recognizable RelA homologs, especially in the higher metazoa, such as mammals [135,136,151]. However, as stated in the Introduction, there is evidence of barely detectable amounts of (p)ppGpp in some metazoa, but mostly in the evolutionarily earlier species, which makes it tempting to speculate that the early metazoa were at the transition from the (p)ppGpp- to NADPH-based response. Studies of more organisms in the boundary between prokaryotes and primitive eukaryotes may unearth various stages of evolution of the stringent response.

The structural similarity between ppGpp and NADPH, particularly in the purine nucleotide parts, has led to the suggestion of their functional equivalence [135]. Both alarmones were/are produced by enzymatic conversion of existing compounds, namely GTP/GDP and NADP/NADPH, which are indispensable for cellular housekeeping functions. In contrast, our knowledge of the role of their interactome is woefully incomplete, as most polypeptides in this group are of unknown function. It is unlikely that all of them simultaneously acquired the ability to bind an alarmone; a more plausible scenario is gradual evolution of small subsets of proteins, initially harboring primordial binding domains that were perfected for optimal activity over time (Figure 8). The previously mentioned evidence that small basal levels of (p)ppGpp have important functions [30,152] and that the affinity of (p)ppGpp-binding proteins can vary over three orders of magnitude, i.e., from micro- to milli-molar concentrations of (p)ppGpp [31,32,38,93,116], suggest that the binding pockets that occur in modern (extant) cells have been stabilized for their respective K_m_ at this stage of evolution. Evolutionary changes in such domains clearly did not affect the housekeeping functions of the proteins, and in fact, they were favorably selected in evolution during exposure to increasingly severe nutrient deprivation. In such a gradually changing environment, the network of the interactome, therefore, increased in size and complexity in punctuated steps, reaching equilibrium at each step. This was also suggested previously for the evolution of thermotolerant organisms, selected during global warming [153].

Of note, there are several other non-nucleic acid nucleotides that are diverse in synthesis, structure, and function. Major examples include: ppApp, which is the adenine counterpart of (p)ppGpp; cyclic nucleotides, such as cAMP, the first recognized nucleotide second messenger; the composite cyclic-GMP-AMP or cGAMP, made from GTP and ATP. Each second messenger acts as a ligand to activate its own interactome of proteins that regulate pathway-specific signaling cascades, triggering specific physiological effects. However, it is not known if they complement or compete with each other, at least for a subset of functions, but such questions have started to draw attention [154]. Regardless, the fundamental molecular concepts discussed here can also apply to all of them.

As the metazoa perfected intercellular communications, such as cellular adhesins, hormones, immunity, and neural networks, they acquired the ability to manipulate the environment, and migrate and mitigate the nutritional shortages, which largely obviated the need to maintain the (p)ppGpp-based stringent response. It was replaced by the NADPH-based system, whose interactome might have evolved through analogous pathways (Figure 8). It is conceivable that the dephosphorylation of NADPH by MESH1 would inhibit the biosynthesis of a variety of compounds that are dispensable in limiting conditions, such as fatty acids and cholesterol, normally required for the formation of new cell membranes during cell division [155], thus conserving the anabolic resources.

## 6. Conclusions

A fundamental tenet of biological evolution is survival of the fittest, which becomes particularly important in challenging conditions that would otherwise eliminate the organism. The stringent response and other mechanisms of defense against stress allow survival during such challenges. Both the bacterial stringent response and the recently discovered ‘stringent-like response’ in metazoa are orchestrated by nucleotide derivatives, namely (p)ppGpp and NADPH. The unique mechanism of the ‘stringent-like response’ holds the potential to open fascinating novel pathways of the role of NADPH. The mechanism by which these ‘second messenger’ molecules regulate the large number of proteins they interact with will continue to be a challenging area of future research. Finally, why these and several other second messengers (e.g., cAMP, ppApp, cGAMP) are all nucleoside phosphates also remains a biochemical and evolutionary enigma.

## Data Availability

No new data were created. The resources and their sources have been fully described in the paper.

## Supplementary Materials

The supporting information can be downloaded at: https://www.mdpi.com/article/10.3390/ijms24043999/s1.
