# Supplementary material for: Protein–Ligand Interactions in Scarcity: The Stringent Response from Bacteria to Metazoa, and the Unanswered Questions
Source: Int J Mol Sci. 2023 Feb 16;24(4):3999. doi: 10.3390/ijms24043999 (PMC9965611; doi:10.3390/ijms24043999)
Supplement: Supplementary file 1 [file ijms-24-03999-s001.zip › ijms-2149920-supplementary.pdf]

Supplementary Material

In silico protein-ligand docking by CB-Dock2 (Relevant to Figures 3,4,5,6):

All protein-ligand structures in this paper were generated in silico, using available crystal structures, deposited in the public domain RCSB PDB (Research Collaboratory for Structural Bioinformatics Protein Data Bank) at www.rcsb.org. The docking was performed with CB-Dock2, which is a recently improved version of its first release. It is a "blind" docking program (i.e. capable of structural cavity prediction, if needed, combined with ligand-fitting tryouts) that is widely used for protein-ligand docking, many with pharmacological success [1]. It is a template-based docking engine with enhanced accuracy in binding-site identification and pose (orientation) prediction, generally with RMSD <2.0. CB-Dock2 was chosen for several features that were relevant to us: easy-to-use web interface; computation speed, the average result return time being about a minute; high accuracy, compared to other "blind" methods, as tested by the authors [1]; multiple parameters offered in the output, such as center, size and volume of the predicted cavities. The use of the web server [1] is free and available at https://cadd.labshare.cn/cb-dock2/. In all our studies in this paper, we used the "structure-based docking" link in the web site, since we were employing protein 3D structures that were already determined. The site accepts the protein structure in PDB format, and the ligand (e.g. ppGpp) in PDB, sdf, and mol2 formats, although we used the mol2 format only. We followed the two-step commands: Search Cavities, then Auto Blind Docking. The returned output generally consisted of multiple structures in which the ligand was docked into cavities with a range of binding energies, from which we selected the one with the strongest binding (negative kcal/mol). For example, in docking the ppGpp mol2 structure into *B. subtilis* Obg, PDB 1LNZ (Figure 3), five different structures were received with energy values of -9.3, -9.1, -8.9, -8.8, and -8.6 kcal/mol, among which we selected the -9.3 kcal/mol structure, and as described in Section 3.2, it was in good agreement with the structure previously reported by Kanjee et al. [2], thus offering confidence.

1. Liu, Y.; Yang, X.; Gan, J.; Chen, S.; Xiao, Z.-X.; Cao, Y. CB-Dock2: improved protein-ligand blind docking by integrating cavity detection, docking and homologous template fitting. *Nucleic Acids Res.* **2022**, *50(W1)*:W159-W164. DOI: 10.1093/nar/gkac394 (same as Ref 96 in the main paper).
2. Kanjee, U.; Ogata K.; Houry, W.A. Direct binding targets of the stringent response alarmone (p)ppGpp. *Mol. Microbiol.* **2012,** *85,* 1029-1043. DOI: 10.1111/j.1365-2958.2012.08177.x (Same as Ref 31 in the main paper).

Sequence similarity between the Ornithine Decarboxylase (ODC) of *Salmonella typhimurium* (Styphi) and *Trypanosoma brucei gambiense* (Tbrucei) (Relevant to Section 3.4.1 and Figure 4)"

The amino acid sequences correspond to PDB 6N2H and PDB 1NJJ, respectively, since these 3D structures were used in Figure 4 . Sequence alignment was performed in Clustal Omega (www.ebi.ac.uk); when all the identical and conserved residues (asterisk *, colon : and full stop/ period .) were added (total 242 residues out of 471), the two sequences were 51% similar.

```
Styphi_ODC      MTDSIMQNYNQLREQVINGDRRFQHKDG-----HLCFEGVDLDALARQYPTPFYVFSEPE    55
Tbrucei_ODC     ----------KSMDIVVNDDLSCRFLEGFNTRDALCKK---ISMNTCDEGDPFFVADLGD    47
                          :  : *:*.*   :. :*      ** :   :.  : :   **:* .  :

Styphi_ODC      IIRNIHEIQQAFAAHKNTKTFFASKT---CSVMGVLKAIRDAGICAEANSQYEVRKCLEI    112
Tbrucei_ODC     IVRKHETWKKCLP---RVTPFYAVKCNDDWRVLGTLAAL---GTGFDCASNTEIQRVRGI    101
                *:*: .  ::.:    ... *:* *      *:*.* *:   *   :. *: *:::   *

Styphi_ODC      GFRGDQIVFNGVVKKPADLEYAIANDLYLINVDSLYELEHIDAISRKLKKVANVCVRVEP    172
Tbrucei_ODC     GVPPEKIIYANPCKQISHIRYARDSGVDVMTFDCVDELEKVAKTHPKAKMVL----RIST    157
                *.  ::*:: .  *: :.:.**  ..: ::..*.: ***::     * * *     *:.
```

```
Styphi_ODC       NVPSATHAELVTAFHAKSGLDLEQAEETCRRILAM---PYVHLRGLHMHVGDQVPESEPF    229
Tbrucei_ODC      DD-SLAR--------CRLSVKFGAKVEDCRFILEQAKKLNIDVTGVSFHVGSGSTDASTF    208
                 :  * ::        .: .:.:    * ** **       :.: *: :***.   ::. *

Styphi_ODC       AKATKVLVDESRRLEEVLGIKFDLINVGGGIPVPYKYDDENGDPLKDNMYAGITAQDFAD    289
Tbrucei_ODC      AQAISDS-RFVFDMGTELGFNMHILDIGGGFPGTR------DAPLKFEEIAGVINNALEK    261
                 *:* .        :   **:::.::::***:*         . *** :  **:  : : .

Styphi_ODC       AVIREVHKWRTDVEICIEPGRKVTGSAAVLLTEVSCEKRKTNYDLNGNVECHVEWK----    345
Tbrucei_ODC      H-----FPPDLKLTIVAEPGRYYVASAFTLAVNVIAKKVTP--GVQTDVGAHAESNAQSF    314
                       .    .: *  ****  ..** .* .:* .:* .   .:: :* .*.* :

Styphi_ODC       --FVDAGYSVLSDSQHFDWFFYVYNASRM-------TAAHDAWIKLAGPLCDGGDYFHMG    396
Tbrucei_ODC      MYYVNDGV-----YGSFNCILYDHAVVRPLPQREPIPNEKLYPSSVWGPTCDGLDQIV--    367
                   :*: *         *: ::* : . *           :    .: ** *** * :

Styphi_ODC       VKGEEFLLPKETHVGDIVAFLDAGAYTIESQTVYNNRPRTGVVMIDKNGDTRLIRREDSY    456
Tbrucei_ODC      ---ERYYL-PEMQVGEWLLFEDMGAYTVVGTSSFNGFQSPTIYYVVSGLPDHVVRELKSQ    423
                    *.: *  * :**: : * * ****: . : :*.     :  : ..   :::*. .*

Styphi_ODC       EDMVKYDIYLAAALE    471
Tbrucei_ODC      KS-------------    425
                 :.
```
